# Supplementary material for: Individual Differences in the Neurocognitive Effect of Movement During Executive Functioning in Children with ADHD: Impact of Subtype, Severity, and Gender
Source: Brain Sci. 2025 Jun 9;15(6):623. doi: 10.3390/brainsci15060623 (PMC12190672; doi:10.3390/brainsci15060623)
Supplement: Supplementary file 1 [file brainsci-15-00623-s001.zip › Table S2.pdf]

Table S2. fNIRS general linear model (GLM) contrast results for ADHD severity analyses

Severity	Participant ID	DLPFC ROI	HbO			HbR		
			β	T-value	P-value	β	T-value	P-value
		F1	-0.44	-4.93	<.001	1.01	11.32	<.001
**Low**	2	F3	0.55	5.45	<.001	-0.22	-15.14	<.001
		F5	0.14	10.36	<.001	0.44	-9.06	<.001
		F1	-0.33	-2.09	.03	-0.13	5.43	<.001
**Low**	28	F3	-0.26	-8.65	<.001	0.61	0.57	.56
		F5	0.60	-24.6	<.001	-0.32	1.92	.05
		F1	0.19	-5.28	<.001	-0.07	-0.61	.53
**Low**	32	F3	0.18	-2.86	.004	-0.48	-17.54	<.001
		F5	0.36	-0.89	.38	0.42	-6.04	<.001
		F1	-0.05	-4.65	<.001	-0.32	30.04	<.001
**Low**	41	F3	0.20	-10.6	<.001	-0.30	26.55	<.001
		F5	0.86	-18.7	<.001	-0.59	0.35	.71
		F1	0.92	-0.55	.57	-0.82	-11.11	<.001
**Low**	29	F3	0.08	8.16	<.001	-0.03	-19.78	<.001
		F5	0.23	-10.57	<.001	-0.09	-1.38	.16
		F1	0.85	-23.92	<.001	-0.79	9.63	<.001
**Low**	53	F3	1.11	-9.21	<.001	-1.21	-5.23	<.001
		F5	0.91	-17.1	<.001	-0.68	2.78	.01
		F1	0.21	-11.84	<.001	-0.43	11.66	<.001
**Low**	9	F3	-0.09	-13.27	<.001	-0.02	12.31	<.001
		F5	0.49	-7.3	<.001	-0.31	-.15	.88
		F1	-0.16	2.02	.04	-0.40	10.87	<.001
**Moderate**	36	F3	0.12	24.42	<.001	-0.37	-1.89	.05
		F5	0.63	-10.14	<.001	0.21	-0.76	.44
		F1	1.51	-5.21	<.001	0.17	19.25	<.001
**Moderate**	51	F3	1.08	-10.77	<.001	-1.07	33.08	<.001
		F5	1.48	-1.41	.16	-0.92	-7.54	<.001
		F1	-1.00	-1.91	.05	0.97	-14.27	<.001
**Moderate**	39	F3	-0.25	-0.07	.94	0.54	-8.89	<.001
		F5	-0.13	0.85	.39	0.66	4.95	<.001
		F1	0.67	-9.21	<.001	0.96	8.63	<.001
**Moderate**	52	F3	-0.30	8.91	<.001	0.79	16.03	<.001
		F5	-0.91	-6.53	<.001	1.30	5.47	<.001
		F1	0.27	-0.51	.6	0.82	-1.53	.12
**Moderate**	30	F3	0.19	-1.99	.05	0.68	12.81	<.001
		F5	-0.18	-10.72	<.001	-0.21	16.34	<.001
		F1	1.21	-8.2	<.001	-0.77	-13.21	<.001
**Moderate**	49	F3	0.20	15.96	<.001	-0.85	17.9	<.001
		F5	0.10	-6.29	<.001	-0.88	3.5	.001
		F1	0.17	-4.24	<.001	1.15	-11.52	<.001
**Moderate**	17	F3	-0.27	-6.65	<.001	0.69	-8.12	<.001

		F5	0.62	7.68	<.001	0.07	-12.82	<.001
		F1	0.05	1.19	.23	0.27	-1.09	.27
**Moderate**	24	F3	0.01	6.61	<.001	0.33	4.96	<.001
		F5	0.26	5.71	<.001	0.51	16	<.001
		F1	-0.54	-1.5	.12	0.20	32	<.001
**Moderate**	27	F3	-0.67	.86	.38	0.72	16	<.001
		F5	-0.70	-3.25	.001	-0.09	-3.8	<.001
		F1	0.19	9.31	<.001	-0.62	13.93	<.001
**Moderate**	5	F3	0.25	9.84	<.001	-0.35	-.12	.90
		F5	-0.20	6.01	<.001	-0.47	1.61	.11
		F1	-0.12	17.01	<.001	0.18	11.12	<.001
**Severe**	14	F3	-0.57	12.76	<.001	-0.01	14.41	<.001
		F5	-0.34	-7.14	<.001	0.39	9.21	<.001
		F1	-0.17	-5.36	<.001	-0.53	1.14	.25
**Severe**	31	F3	-0.31	-7.27	<.001	-0.17	-8.08	<.001
		F5	-0.96	10.08	<.001	0.62	-9.27	<.001
		F1	0.96	14.62	<.001	0.20	-14.01	<.001
**Severe**	35	F3	-0.32	2.12	.03	-0.58	4.93	<.001
		F5	0.89	20.55	<.001	-0.42	-17.88	<.001
		F1	-0.39	14.63	<.001	0.05	4.09	<.001
**Severe**	40	F3	-0.31	7.76	<.001	-0.02	2.53	.01
		F5	0.57	-10.16	<.001	-0.03	12.52	<.001
		F1	-0.38	-8.23	<.001	-1.45	-7.21	<.001
**Severe**	42	F3	-0.09	-3.78	<.001	1.41	16.59	<.001
		F5	-1.44	-10.64	<.001	0.72	-4.74	<.001
		F1	0.31	-11.11	<.001	0.32	-1.64	.1
**Severe**	47	F3	0.34	-1.97	.05	0.24	7.94	<.001
		F5	0.68	-6.46	<.001	-0.05	16.41	<.001
		F1	-0.54	12.71	<.001	0.20	-5.75	<.001
**Severe**	13	F3	-0.59	18.36	<.001	-0.34	3.16	.002
		F5	-0.04	25.12	<.001	-0.41	-7.6	<.001
		F1	-0.65	9.51	<.001	0.57	-5.8	<.001
**Severe**	37	F3	-0.71	2.23	.02	0.89	-26.33	<.001
		F5	-0.38	12.74	<.001	0.55	-26.08	<.001
		F1	0.21	-21.78	<.001	1.15	-1.86	.06
**Severe**	3	F3	0.43	5.4	<.001	1.00	-.25	.80
		F5	0.93	-13.09	<.001	-0.17	-11.33	<.001

Note. HbO (oxygenated hemoglobin), HbR (deoxygenated hemoglobin), DLFPC (dorsolateral prefrontal cortex), ROI (region of interest), F1 (fNIRS channel source 4-detector 2), F3 (fNIRS channel source 1-detector 2), F5 (fNIRS channel source 1-detector 1), β (Beta coefficient, effect size).
